# COVID-19 severity determinants inferred through ecological and epidemiological modeling

**DOI:** 10.1016/j.onehlt.2021.100355

**Published:** 2021-11-27

**Authors:** Sofija Markovic, Andjela Rodic, Igor Salom, Ognjen Milicevic, Magdalena Djordjevic, Marko Djordjevic

**Affiliations:** a Quantitative Biology Group, Faculty of Biology, University of Belgrade, Serbia; b Institute of Physics Belgrade, National Institute of the Republic of Serbia, University of Belgrade, Serbia; c Department for Medical Statistics and Informatics, School of Medicine, University of Belgrade, Serbia

**Keywords:** COVID-19, Disease severity, Ecological regression analysis, Epidemiological model, Environmental factors, Machine learning

## Abstract

Understanding variations in the severity of infectious diseases is essential for planning proper mitigation strategies. Determinants of COVID-19 clinical severity are commonly assessed by transverse or longitudinal studies of the fatality counts. However, the fatality counts depend both on disease clinical severity and transmissibility, as more infected also lead to more deaths. Instead, we use epidemiological modeling to propose a disease severity measure that accounts for the underlying disease dynamics. The measure corresponds to the ratio of population-averaged mortality and recovery rates (*m*/*r*), is independent of the disease transmission dynamics (i.e., the basic reproduction number), and has a direct mechanistic interpretation. We use this measure to assess demographic, medical, meteorological, and environmental factors associated with the disease severity. For this, we employ an ecological regression study design and analyze different US states during the first disease outbreak. Principal Component Analysis, followed by univariate, and multivariate analyses based on machine learning techniques, is used for selecting important predictors. The usefulness of the introduced severity measure and the validity of the approach are confirmed by the fact that, without using prior knowledge from clinical studies, we recover the main significant predictors known to influence disease severity, in particular age, chronic diseases, and racial factors. Additionally, we identify long-term pollution exposure and population density as not widely recognized (though for the pollution previously hypothesized) significant predictors. The proposed measure is applicable for inferring severity determinants not only of COVID-19 but also of other infectious diseases, and the obtained results may aid a better understanding of the present and future epidemics. Our holistic, systematic investigation of disease severity at the human-environment intersection by epidemiological dynamical modeling and machine learning ecological regressions is aligned with the One Health approach. The obtained results emphasize a syndemic nature of COVID-19 risks.

## Highlights

• Ecological and epidemiological modeling are combined to analyze COVID-19 severity.
• A new severity measure, independent of transmissibility, is proposed.
• Determinants of COVID-19 clinical severity are inferred by machine learning.
• Chronic disease prevalence, age and race, are reproduced as clinically known risks.
• Pollution and population density, underscoring complex risks, are also obtained.

## 1 Introduction

COVID-19 has brought large changes to people's lives, including significant impacts on health and the economy. At the population level, the effects of the disease can be characterized through the disease transmissibility and clinical severity. Transmissibility relates to the number of infected people, which in epidemiological models (see e.g. [1]) is quantified by the reproduction number *R*(*t*) (corresponding to an average number of people infected by an individual during its infectious period). Clinical severity corresponds to the medical complications experienced by infected individuals, potentially also including death. In the epidemic models, two population average rates relate with the disease severity (see e.g. [2]): *i*) Mortality rate (*m*), corresponding to the probability per day for the detected case to result in death. ii) Recovery rate (*r*), corresponding to the inverse time needed for a detected case to recover.

This study aims to quantify the generalized severity of the COVID-19 disease in a given population described with a set of demographic and environmental factors by proposing an easy-to-evaluate but plausible measure. Applying that measure to diverse countries, together with carefully designed multivariate regression analysis, allows identifying the severity predictors among the population-level factors. Knowing the particular causes of a potentially higher fatality of the disease in a population can help plan efficient strategies for disease prevention, control, and treatment, specifically targeting the health vulnerabilities of the society.

COVID-19 transmissibility and severity are often assessed through the numbers of confirmed cases and fatalities, respectively [[3], [4], [5], [6], [7], [8]]. Regarding severity, a major complication is that the fatalities are correlated with the number of infected, as more infections lead to more fatalities. Additional complications are related to nonlinearities and delays that inherently characterize the disease dynamics [9]. For example, the time from infection to death can be long and highly variable, while the number of fatalities in different regions (at a given time) may correspond to different points of the infected curve. Thus, equal-time comparisons of mortality numbers (or rates) would be inadequate. For these reasons, somewhat modified variables, such as delay-adjusted case fatality rate (aCFR), are sometimes used [[10], [11], [12]], but their mechanistic interpretation is unclear [13]. Alternatively, we propose a novel quantity for the disease severity measure. This quantity has a clear mechanistic interpretation, can be derived directly from epidemic modeling and inferred from publically available data. Specifically, we argue that the ratio of mortality and recovery rates (*m*/*r*) is a highly plausible population-level measure of disease severity: higher mortality and lower recovery rates indicate a more severe disease leading to a larger *m*/*r*. We will also show (both theoretically and from empirical data) that this measure is a priori unrelated to *R*(t), which is a result independent from the specific assumed transmission mechanism.

To assess how reasonable is the proposed measure, it is desirable to use it to infer significant predictors (and their importance) of COVID-19 severity. However, this entails certain methodological challenges [14]. Specifically, significant predictors have to be selected among a large number of potentially relevant variables. Moreover, these variables may be highly correlated [15,16], and mutual interactions (and nonlinear relations) may be relevant. To address this, we apply a novel approach that combines Principal Component Analysis (PCA) and machine learning regression methods [17].

## 2 Methods

**Fig. 1:**
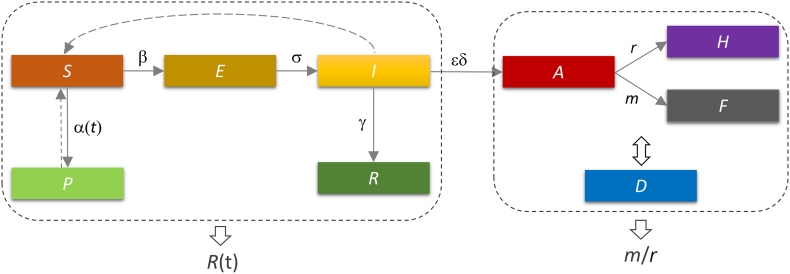
Deriving the severity measure *m*/*r* from the epidemics compartmental model. SPEIRD model is schematically shown. Transitions between the compartments are denoted by solid arrows, with the transition rates indicated above arrows. The dashed arrow from I to S indicates the interaction of I and S (infections) leading to the transition to E. The dashed arrow from P to S indicates the potential (reverse) transition from P to S due to the easing of measures. The dashed rectangles indicate parts of the model corresponding to the disease transmission (the left rectangle) and the disease outcome for the detected cases (the right rectangle). The single arrows indicate parts of the model from which the reproduction number *R*(t) and the severity measure (*m*/*r*) are, respectively, inferred. The total number of detected cases (D) corresponds to the sum of A, H, and F and is denoted by a double arrow. Compartments are S – susceptible, P –protected, E – exposed, I –infected, R – recovered, A – active, H – healed, F – fatalities, D – total number of detected cases. *r* and *m* represent recovery and mortality rates of active (detected) cases.

There are many compartmental models used in epidemiology, obtained as extensions of the basic SIR or SEIR models [2] – a number of them recently developed in the context of COVID-19, e.g. to account for contact tracing and hospitalization strategies [18], media effects [19], unreported cases [1,20], infected but asymptomatic individuals [21], uninfected but quarantined population [22], seasonality effects [23], etc. To extract the severity variable *m*/*r* directly from dynamical compartmental models, we used our SPEIRD modification [24] of the SEIR model, schematically represented in Fig. 1. Note that *m*/*r* derivation is independent of the transmission mechanism and (by construction) from *R*(*t*). Consequently, the left rectangle (from which *R*(t) and its special case at the early stages of the epidemic, i.e., basic reproduction number (*R*_0_), is determined) is presented only for clarity and coherence. The relevant part of the model represents the transition of the active cases (*A*) to healed (*H*) at recovery rate *r*, or to fatalities (*F*) at mortality rate *m*. Note that the cumulative (total) number of detected cases (*D*) corresponds to the sum of *A*, *H*, and *F*.

The system of differential equations, which mathematically represents the model in Fig. 1 is given in [24]. From eqs. (5–6) in [24], we obtain:

(1) $$\frac{\mathrm{dH}}{\mathrm{dt}}=rA;\frac{\mathrm{dF}}{\mathrm{dt}}=mA\Rightarrow \frac{\mathrm{dF}}{\mathrm{dt}}=\frac{m}{r}\frac{\mathrm{dH}}{\mathrm{dt}}$$

We integrate the right side of Eq. (1) from the epidemics start (*t* = 0) to the end (t = ∞):

(2) $$\begin{array}{c} F\left(\infty \right)=\frac{m}{r}H\left(\infty \right) \end{array}$$

**Fig. 2:**
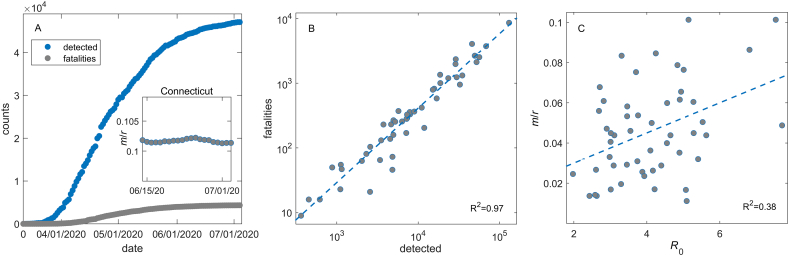
Inferring *m*/*r* from data. A) Cumulative detected (D) and fatality (F) counts in Connecticut. *m*/*r* is inferred from the time period (enlarged in the inset) corresponding to saturation (end of the first peak). B) and C) Correlation plots of F vs. D and *m*/*r* vs *R*_0_ with the Pearson correlation coefficients shown.

Since *D*(*t*) = *A*(*t*) + *F*(*t*) + *H*(*t*), and since there are no more active cases at t = ∞, while *F*(∞) and *H*(∞) reach constant values (see Fig. 2A), we obtain:

(3) $$\begin{array}{c} D\left(\infty \right)=F\left(\infty \right)+H\left(\infty \right) \end{array}$$

Combining Eqs. (2) and (3) gives:

(4) $$\begin{array}{c} \frac{m}{r}=\frac{\mathrm{CFR}\left(\infty \right)}{1-\mathrm{CFR}\left(\infty \right)};\mathrm{CFR}=\frac{F\left(\infty \right)}{D\left(\infty \right)} \end{array}$$

where CFR(∞) is the case fatality rate at the end of the epidemic (the “long COVID-19” cases, being detected but not dead, contribute to *H*(∞) in Eq. (3)). As the COVID-19 pandemic is still ongoing, we use the end of the first peak, where the number of active cases can be approximately considered as zero.

For consistency and easier direct comparison with the COVID-19 transmissibility analysis, data collection, data processing, and machine learning techniques are similar to the one presented in [25]. For completeness, full information is provided in the Supplementary Methods, which also includes definitions for all variables and principal components (PCs) used in the analysis. Supplementary Methods also provide a complete dynamical model and derivations for both *m/r* and *R*_*0*_. The Supplementary Table contains all input data.

## 3 Results

Fig. 2A illustrates how *m*/*r* values are inferred from data. The cumulative number of detected cases and fatalities during the first peak of the epidemic is presented for one of the USA states (Connecticut). *m*/*r* is inferred once both classes of the case counts reach saturation, leading to constant *m*/*r* (inset in the figure). In Fig. 2B a very high positive correlation (*R* = 0.97) is obtained between the cumulative number of fatalities and detected cases observed at fixed time cross-sections, quantitatively confirming the intuitive expectation that a higher number of infected is strongly related to higher fatality counts. This shows that fatality counts are strongly related to COVID-19 transmissibility, despite being often used as a measure of the disease severity [[3], [4], [5], [6], [7], [8]]. On the other hand, the moderate correlation between *m/r* and *R*_0_ (Fig. 2C) is consistent with the a priori independence of these two variables. This correlation reflects a genuine similarity in COVID-19 transmissibility and severity determinants (e.g., air pollution or weak immunity can be associated with both increased transmissibility [25] and severity of the disease [26]).

**Fig. 3:**
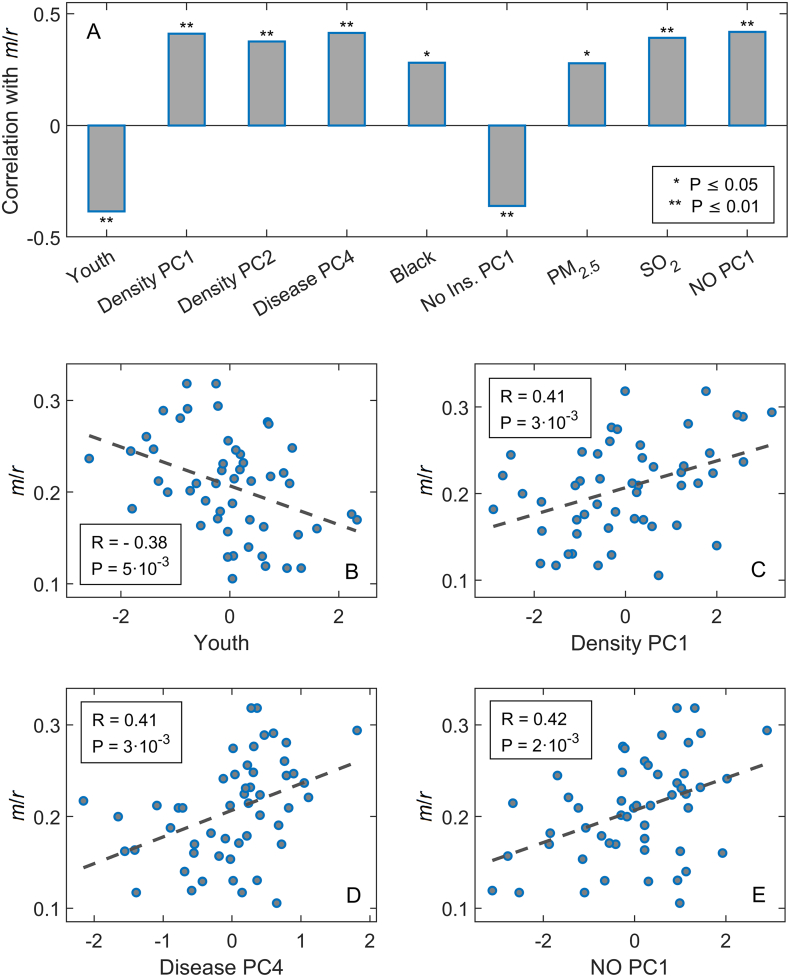
Univariate correlation analysis. (A) Values of Pearson's correlations for the variables significantly correlated (*P* < 0.05) with *m*/*r*. Correlation plots of *m*/*r* with (B) Youth (percent of the population under 18), (C) density PC1, (D) disease PC4, (E) NO PC1.

Univariate analysis of *m*/*r* relation to the variables used in the study is presented in Fig. 3. Statistically significant correlations (*P* < 0.05) of *m*/*r* with several variables/PCs are shown in Fig. 3A and scatterplots (Fig. 3B-E). The highest (positive) correlation was observed for NO PC1, Disease PC4, and Density PC1, while the percentage of the youth population showed the highest negative correlation with *m*/*r*. Several other predictors, specifically, Density PC2, Disease PC2, SO_2_, and NO Insurance PC1, Black and PM_2.5_ also exhibit statistically significant correlations with *m*/*r*. As expected, chronic disease, pollution, population-density-related variables promote COVID-19 severity (positive correlations), as does the percentage of Afro-Americans (Black). Percentage of population under 18 (Youth) decreases the severity (negative correlation), also as expected. Sign of the correlation with No Insurance PC1 is opposite than expected, as people with health insurance should get better medical treatment (further analyzed below).

**Fig. 4:**
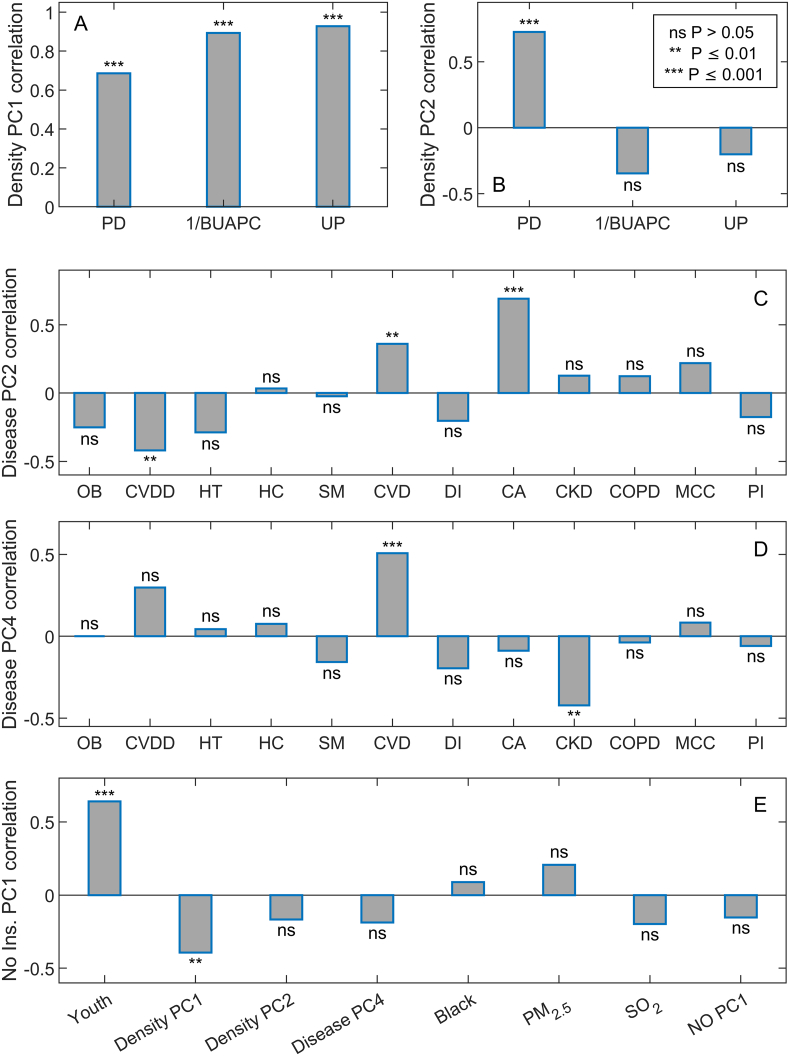
Interpretation of the relevant PCs. Correlation of the relevant principal components with the independent variables is shown, where the height of bars corresponds to the value of Pearson's correlation coefficients. A) and B) Correlation of Density PC1 and Density PC2 with three population density variables. C) and D) Correlation of Disease PC2 and Disease PC4 with the variables from the chronic disease group. (E) Correlations of No Insurance PC1 with the variables from Fig. 3A. The abbreviations correspond to PD – population density, BUAPC – Built-Up Area Per Capita, UP – Urban Population, OB – obesity, CVDD – cardiovascular disease deaths, HT – hypertension, HC – high cholesterol, SM – smoking, CVD – cardiovascular disease, DI – diabetes, CA – cancer, CKD – chronic kidney disease, COPD – chronic obstructive pulmonary disease, MCC – multiple chronic conditions, PI – physical inactivity.

Fig. 4A-D provide interpretation of the relevant PCs through their correlations with the variables entering PCA. Density PC1 is comprised of all three parameters from the population density group (Fig. 4A), presenting a general measure of population density, while Density PC2 is significantly correlated only with population density (Fig. 4B). Disease PC2 and PC4 show, respectively, the highest positive correlation with the prevalence of cancer and cardiovascular diseases. Fig. 4E shows a high correlation of No Insurance PC1 with Youth and Density PC1. Signs of these correlations, and the effect of these two variables on *m*/*r*, indicate that the unintuitive sign of No Insurance PC1 correlation with *m*/*r* (noted above) is an artifact of its high correlations with Youth and Density PC1.

We next performed multivariate analyses, where the effect of each variable on *m*/*r* is controlled by the presence of all other variables. We used Lasso and Elastic net methods [17] that both perform feature selection by shrinking the coefficients of variables that do not affect *m*/*r* to zero, followed by, so-called, relaxed Lasso and Elastic net procedures (as described in Supplementary Methods).

**Fig. 5:**
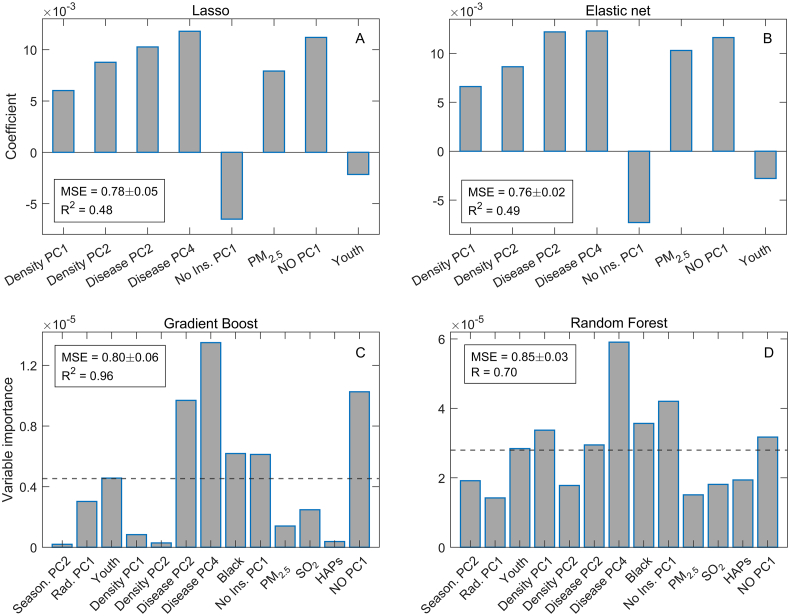
Multivariate (machine learning) analysis. Values of regression coefficients in relaxed A) Lasso and B) Elastic Net regressions. Only the variables whose coefficients are not shrunk to zero by the regressions are shown. The height of bars corresponds to the value of coefficients. Variable importance in C) Gradient Boosting and D) Random Forest regressions, where the height of bars corresponds to estimated importance. Testing set MSE values with the standard errors are shown for each model, corresponding to 5-fold cross-validations with 40 repartitions. Coefficients of determination on the entire dataset (R^2^) are also shown for each model. Variable names are indicated on the horizontal axis.

Both methods robustly show similar results (Fig. 5A-B) and prediction accuracy (MSE indicated in figures). Disease PC4 appears in regressions as the most important predictor, followed by NO PC1 and Disease PC2. Other selected predictors are Density PC1 and PC2, No Insurance PC1, PM_2.5_, and Youth. These results agree with pairwise correlations, except for SO_2_ and Black, which appeared significant in pairwise correlation but were not selected by either linear regression-based method.

We next apply Gradient Boost and Random Forest [17] (see Supplementary Methods), which are non-parametric machine learning methods, i.e., account for potentially highly non-linear relations and interactions between the predictors. For each of these methods, the predictor importance is presented in Fig. 5C-D, where the dashed lines indicate a standard threshold for distinguishing important predictors. Largely consistent results are obtained by both methods, where the predictors with the highest importance are Disease PC4, NO PC1, Disease PC2, No Insurance PC1, Black, and Youth. The only difference is in Density PC1, which appears as important in Random Forest but not in Gradient Boost. Results of Gradient Boost and Random Forest are also consistent with those of Lasso and Elastic Net, with an exception in Black (important in non-linear, but not linear, methods) and PM_2.5_ (vice versa). The effect of Black on *m*/*r* may therefore be nonlinear and/or based on interactions with other predictors (further discussed below).

To test our assumption that No Insurance PC1 appears in regressions due to its high correlation to other *m*/*r* predictors (mainly Youth and Density PC1), we next repeated the analysis, this time excluding No Insurance PC1. The results presented in Supplementary Fig. S1 show that removing No Insurance PC1, besides leading to an (expected) increase of importance of Youth and Density PC1 (which are highly correlated with No Insurance PC1), does not significantly alter previously obtained results – confirming our assumption.

**Fig. 6:**
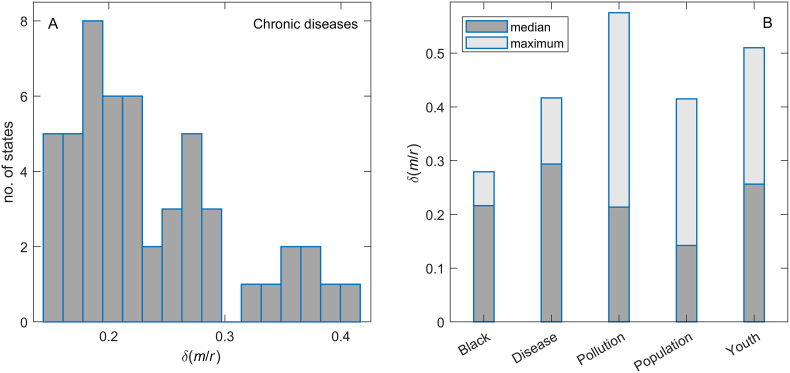
Estimated change in m/r due to variations of important predictors. A) Distribution of relative changes in m/r (δ(m/r)) due to variations in prevalence of chronic diseases observed in USA states. For each state, m/r was predicted for the range of the disease prevalence values observed throughout all other states. B) The same as in A) is repeated, but for the groups of predictors indicated on the horizontal axis. For each group, the median and maximal value of δ(m/r) is reported. δ(m/r) values for each group of predictors are estimated as described in Supplement Methods.

Finally, in Fig. 6, we quantitatively estimate the influence of the five most important predictors determined above. For each of 51 states, we fix the values of all other predictors while changing the analyzed predictor's value within the range observed in all other states. The resulting distribution of the relative changes in *m*/*r* (δ(*m*/*r*)) due to the variation of Chronic disease is shown in Fig. 6A, where each data point in the distribution corresponds to a single USA state. We see that changing Chronic disease values in a realistic range leads to significant variations of *m*/*r*, with a median of ~30% and going up to 40%. To increase robustness, the predictions are made by the consensus of all relevant models. For the remaining four predictors, the obtained median and maximal relative changes are shown in Fig. 6B. The obtained results confirm the importance of Chronic disease, Youth, Black, and Pollution, and, to a smaller extent, Population density.

## 4 Discussion

While we have earlier studied the parameters that might affect *R*_0_ [25,27], the present goal was to investigate which demographic and environmental variables may influence the average disease severity as manifested in a population. The first step was to propose the response variable, which has to be causally independent of *R*_0_ [25,27], to allow understanding the effects of clinical severity alone. We showed that this is indeed satisfied by our choice (*m/r*). Additionally, this work allowed us to mechanistically interpret the standard (simple) measure of clinical severity (CFR), i.e., to relate its saturation value with the rate parameters in the epidemiological dynamical model. The relation is, however, non-linear (sigmoidal), which further underscores the non-triviality of the obtained result.

The proposed measure is practical to implement on a large scale (i.e., for diverse regions or countries, as we here demonstrated for 51 USA states), as only publicly available data are required, and calculation corresponds to a simple (though nonlinear) relation. Estimating the saturation (end of the peak) is straightforward in most cases, through both case counts and *m/r* reaching a saturation (nearly constant) value. We set the following aims for the selected significant predictors of *m/r*: *i*) test if we can recover clinically observed dependencies, *ii*) uncover additional risk factors for COVID-19 clinical severity, suitable to extract from ecological study design [[28], [29], [30]], *iii*) compare with significant predictors of COVID-19 transmissibility (*R*_0_) that we previously obtained [25,27]. We here indeed obtained different predictors for *m/r* compared to *R*_0_ [25,27]. There are also some similarities consistent with inherent connections in COVID-19 transmissibility and severity drivers, e.g., the role of pollution, unhealthy living conditions, and indoor population density [25]. We further discuss *i*) and *ii*).

We obtain that both the prevalence of chronic diseases and Youth significantly influence *m/r*. This is hardly surprising - though quite a non-trivial result - as we started from a large group of initial variables. The influence of Disease PC4, dominantly reflecting the prevalence of cardiovascular diseases, is well documented by clinical studies [31,32] together with some other ecological studies [11,15]. Other chronic conditions that are known COVID-19 comorbidities (i.e., hypertension, obesity, and diabetes) are significant risk factors for cardiovascular diseases [33], and it is not surprising that cardiovascular diseases dominate over other chronic conditions in our results. Disease PC2, dominantly reflecting the prevalence of cancer (though also related to cardiovascular diseases), agrees with CDC warning that people with a history of cancer may be at increased risk of getting severely ill from COVID-19 [34]. Regarding Youth, it is established that younger individuals are, on average, less severely affected by COVID-19, and that the disease severity increases with age [3,35,36].

We found that chronic pollution exposure, NOx levels in particular, significantly promote COVID-19 severity. While difficult to assess through clinical studies, it has been suggested that pollution is associated with the severity of COVID-19 conditions through similar pathways by which it affects respiratory and cardiovascular mortality [37]. In particular, NOx may reduce lung activity and increase infection in the airway [38]. Similarly, the effect of population density (which here significantly affects *m/r*) is hardly suited to detect through clinical studies, while some ecological regression studies also noticed this dependence [39]. An explanation might be that while medical facilities are, in general, more abundant in overcrowded areas [40], this effect becomes overshadowed by the highly increased rate of the COVID-19 spread in these areas. Therefore, population density probably acts as a proxy for smaller healthcare capacity per infected (as the infections increase with the population density, particularly in indoor areas). Additionally, it was also proposed that higher viral inoculum may lead to more severe COVID-19 symptoms [41,42], where overcrowded conditions might lead to higher initial viral doses.

The appearance of the variable Black among the important predictors (Fig. 5C-D) suggests that Afro-Americans are, on average, at higher risk of developing more severe COVID-19. While clinical evidence and several ecological meta-analyses [40,43] seem to confirm this, the underlying reasons are still a matter of debate (see e.g. [44]). Interestingly, this predictor appears only in non-parametric models, where interactions with other predictors are (implicitly) included. A posteriori, this result may not be surprising as it has been argued that higher clinical severity of COVID-19 for Black may be tightly related to other significant factors of COVID-19 severity (larger prevalence of chronic diseases, more crowded conditions, higher air pollution).

Our broad estimates of the magnitude of the effects on *m/r* are also consistent with all four groups of factors (disease, youth/age, pollution, race) being significant drivers of COVID-19 severity, where a somewhat smaller magnitude was obtained for the fifth group (population density). The immediate implications are that prevention of chronic diseases, reduction of pollution, and improving living conditions can indirectly also alleviate the harms of the pandemics.

### 4.1 Limitations

To infer the *m/r* value, our method requires the existence of a well-defined end of an epidemic wave. While all US states met this criterion in the first COVID-19 wave, in general, this may show up as a limitation.

Some of the studied predictors exhibited limited variability across US states. E.g., this must be considered when interpreting the absence of meteorological variables from the set of significant predictors (despite their significant association with *R*_0_ [18,37]) and the presence of air pollution (occasionally hypothesized to contribute to COVID-19 severity [45]).

## 5 Conclusion

We employed a cross-disciplinary (One Health) approach [[46], [47], [48], [49]], combining epidemiological modeling with advanced statistical (machine) learning approaches, to explore the relationship of environmental factors to COVID-19 clinical severity. From a large number of variables, we achieved a robust selection of a small number of significant factors, including those that are clinically known as determinants of COVID-19 severity. Our findings (performed in an unbiased manner directly from the data) are thus consistent with previous clinical studies – which may be interpreted as a kind of experimental validation of our method. Additionally, our results underscore a syndemic nature of COVID-19 risks [50] through a selection of variables related to pollution, population density, and racial factors (intertwined with the effects of other factors). These results might have important implications for both longer and shorter-term efforts to alleviate the effects of this and (likely) future epidemics, in terms of longer-term policies to reduce these risks and shorter-term efforts to accordingly relocate medical resources. Our proposed measure (independent of disease transmissibility) originates from general considerations that are not limited to COVID-19. Thus, it may also be utilized in potential future outbreaks of infectious diseases, possibly also combined with other more traditional measures [9].

## Conflict of interest statement

The authors declare that the research was conducted in the absence of any commercial or financial relationships that could be construed as a potential conflict of interest.

## Appendix A Supplementary data

Supplementary Table: Input data for US states. Image 1

Supplementary Figure S1 Image 2

Supplementary Methods Image 3
