# Supplementary material for: COVID-19 severity determinants inferred through ecological and epidemiological modeling
Source: One Health. 2021 Nov 27;13:100355. doi: 10.1016/j.onehlt.2021.100355 (PMC8626896; doi:10.1016/j.onehlt.2021.100355)
Supplement: Supplementary file 2 — Supplementary Figure S1 [file mmc2.pdf]

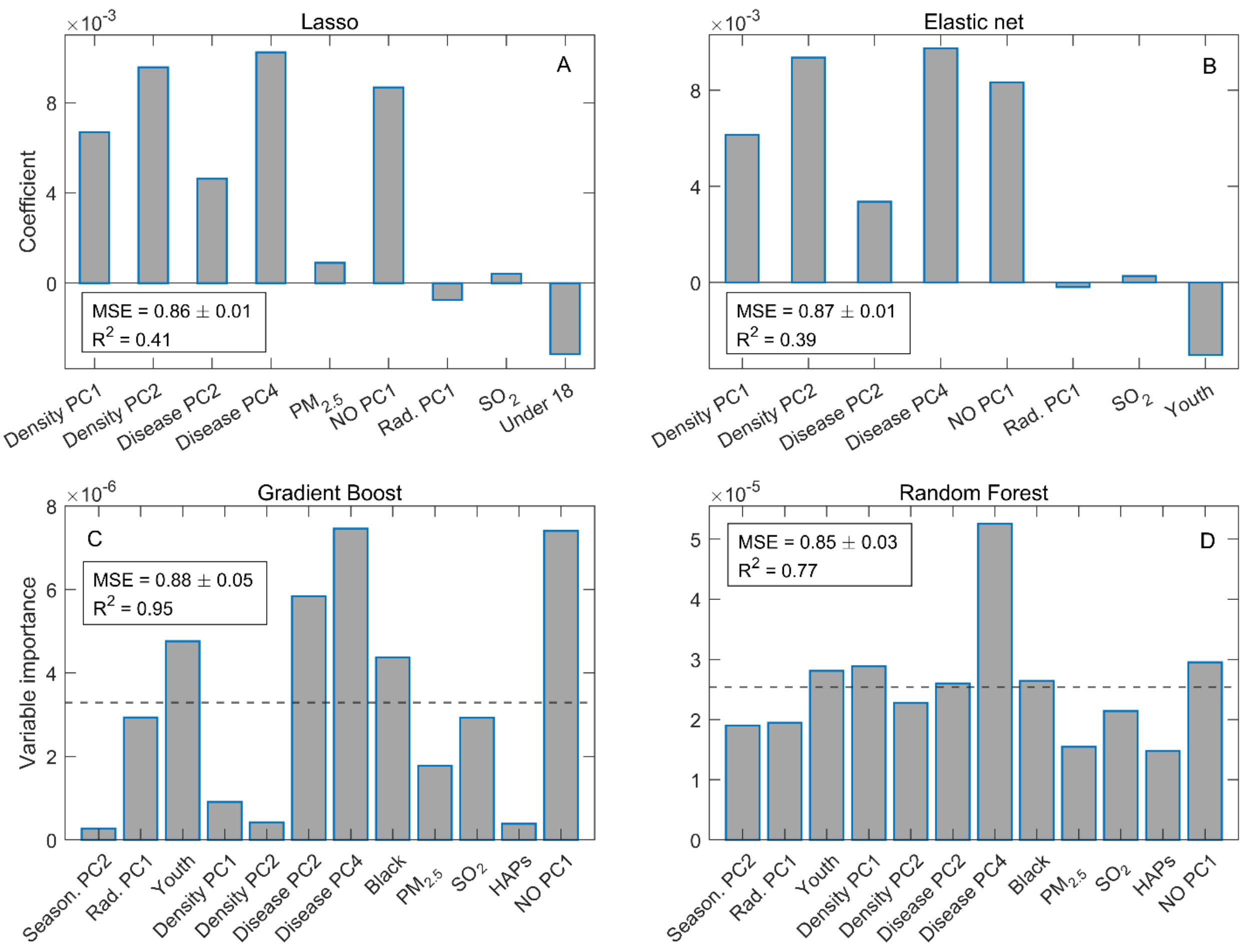

Figure S1: Multivariate (machine learning) analysis without No insurance PC. The equivalent analysis as in Figure 5 is performed, but with excluded No Insurance PC data. The explanation is the same as in the caption of Figure 5.
