# Supplementary material for: COVID-19 severity determinants inferred through ecological and epidemiological modeling
Source: One Health. 2021 Nov 27;13:100355. doi: 10.1016/j.onehlt.2021.100355 (PMC8626896; doi:10.1016/j.onehlt.2021.100355)
Supplement: Supplementary file 3 — Supplementary Methods [file mmc3.pdf]

Supplementary Methods

The SPEIRD model of the COVID-19 epidemic

To extract the basic reproduction number R_0 and the severity variable m/r, we used a modification of an SEIR-based compartmental model [1] introduced in our earlier paper [2], describing the movement of individuals between compartments as observed in the case of the COVID-19 spread through a population (Figure 1). This (SPEIRD) model assumes that a Susceptible (S) individual who encounters an Infected (I) (indicated by the dashed arrow in the figure) first becomes Exposed (E) to the virus and, after some latency time, infectious for other people, moving to the compartment I. By practicing social distancing, Susceptibles move to the compartment P (Protected), with a time-dependent protection rate that in principle can also be reversible corresponding to easing social distancing (represented by the dashed arrow). The Infected individuals either Recover (or die) without being diagnosed, moving to R, or they enter the pool of the Active detected cases (A), from where they can either move to H (Healed), if they recover, or to F (Fatalities), if they succumb to the illness. The sum of the individuals in A, H, and F gives the total number of Detected cases, D.

The model is described by the following system of ordinary differential equations (the size of the population is denoted with N):

$$\frac{dS}{dt} = -\frac{\beta \cdot S \cdot I}{N} - \alpha(t) \cdot S \tag{1}$$

$$\frac{dP}{dt} = \alpha(t) \cdot S \tag{2}$$

$$\frac{dE}{dt} = \frac{\beta \cdot S \cdot I}{N} - \sigma \cdot E \tag{3}$$

$$\frac{dI}{dt} = \sigma \cdot E - \gamma \cdot I - \varepsilon \cdot \delta \cdot I \tag{4}$$

$$\frac{dR}{dt} = \gamma \cdot I \tag{5}$$

$$\frac{dD}{dt} = \varepsilon \cdot \delta \cdot I \tag{6}$$

where Eq. (6) for the total number of detected cases is obtained by summing the equations for the remaining three compartments:

$$\frac{dA}{dt} = \varepsilon \cdot \delta \cdot I - r \cdot A - m \cdot A \tag{7}$$

$$\frac{dH}{dt} = r \cdot A \tag{8}$$

$$\frac{dF}{dt} = m \cdot A. \tag{9}$$

According to the model, the detected cases do not participate in the spreading of the virus, as they are considered to be quarantined. Also, the model assumes that the protection rate (α) changes with time because the social distancing measures were introduced after the initial interval of the uncontrolled epidemic growth. For the currently presented analysis, this part of the model is not needed, while in our

earlier papers we allowed α to increase according to a step [2] or a Hill function [3] from the moment of introduction of the intervention measures in a particular country/region. For simplicity, we here neglect the reversible transition from P to S, as this does not impact the derivation of R_0 and m/r (see below). We constructed this model aiming for a general mathematical description of the COVID-19 epidemic that could be consistently applied to the scenarios of numerous different countries and regions. Therefore, it describes the key, basic features of the disease spread, while additional effects such as demographic, spatial, and other population heterogeneities (network effects) [4,5], and seasonality effects [6] were omitted. Including those effects should provide a finer agreement of the model with the data and a greater prediction power, but at the cost of a larger number of parameters whose values would be hard to determine from the data without over-fitting. On the other hand, our deterministic, compartmental SPEIRD model, as we showed previously [2,3,7–9], provides a reasonable description of the COVID-19 epidemic spread and can be easily related to the common growth regime patterns observed in the cumulative case curves during the first wave for the majority of world countries [3]. These additional effects also do not impact m/r derivation (see below).

In [2,3], we developed and applied the procedure for estimating the values of all model parameters, describing the complete course of the first wave from the epidemic outburst. However, here we do not need it, as we mathematically show that our clinical severity measure, m/r, can be evaluated for a given country/region based only on cumulative cases and fatalities data (see the Supplementary section "Derivation of m/r"). Nevertheless, in a part of the present analysis, we use the R_0 values that we previously determined for different countries, so we outline this derivation in the Supplementary section "Derivation of R_0".

Derivation of R_0

The average number of secondary cases per infectious case in a completely susceptible population is known in epidemiology as the basic reproduction number, R_0. The SPEIRD model was used to analytically derive the equation for R_0 [10,11] - a measure of the SARS-CoV-2 transmissibility in a fully susceptible population and in the absence of intervention measures (social distancing, quarantine). Under these conditions, the model reduces to two linear differential equations:

$$\frac{dE}{dt} = \beta \cdot I - \sigma \cdot E \tag{10}$$

$$\frac{dI}{dt} = \sigma \cdot E - \gamma \cdot I. \tag{11}$$

The solution of the system of Eqs. (10) and (11) take the form

$$I(t) = C_1 \cdot e^{\lambda_+ t} + C_2 \cdot e^{\lambda_- t} \tag{12}$$

where λ_+ and λ_- are the system eigenvalues. The term containing the negative eigenvalue can be neglected, as we showed in [7], leading to

$$I(t) = I(0) \cdot e^{\lambda_+ t}. \tag{13}$$

The basic reproduction number, $R_0 = \beta/\gamma$ [1,12], can be expressed in terms of the positive eigenvalue:

$$R_0 = 1 + \frac{\lambda_+ \cdot (\gamma + \sigma) + \lambda_+^2}{\gamma \cdot \sigma}. \tag{14}$$

By taking the logarithm of the solution of the Eq. (6),

$$D(t) = \varepsilon \cdot \delta \cdot I(0) \cdot (e^{\lambda_+ t} - 1)/\lambda_+, \quad (15)$$

we obtain the equation for the linear dependence of the cumulative number of total detected cases on time on a semi-logarithmic scale:

$$\log\big(D(\mathrm{t})\big) = \log(\varepsilon \cdot \delta \cdot I(0)/\lambda_+) + \lambda_+ \cdot \mathrm{t}\,. \quad (16)$$

Fitting the Eq. (16) to the data for the initial exponential phase of the epidemic growth lasting for ~2 weeks, which can be easily identified observing the curves for a wide range of countries before the onset of the control measures [3], the value of λ_+ is obtained as the slope of the straight line [12]. Thus, the R_0 value can be calculated according to Eq. (14) for fixed, literature values of σ (1/3 day^{-1}) and γ (1/4 day^{-1}). R_0 may be regarded as a special case of the effective reproduction number, R_e, which represents the average number of secondary cases per one infected in a population consisting of both susceptible and non-susceptible individuals. The derivation of the severity measure does not depend on the transmission paths framed in the left part of Figure 1 and is, therefore, independent from $R_e(t)$.

Derivation of m/r

The part of the model which is relevant for the derivation of our clinical severity variable (m/r) is framed on the right in Figure 1 and describes the transition of the active cases (*A*) to *H*, at the recovery rate *r*, or to *F*, at the mortality rate *m*. We integrate both sides of the equation

$$\frac{dF}{dt} = \frac{m}{r} \cdot \frac{dH}{dt}, \quad (17)$$

which follows from Eqs. (8-9), from the start (t = 0) to the end (t = ∞) of the epidemic wave to obtain:

$$F(\infty) = \frac{m}{r} H(\infty). \quad (18)$$

At t = ∞, there are no more actively detected cases, while *F*(*t*) and *H*(*t*) reach constant values (see Fig. 2A), so the total number of detected cases becomes:

$$D(\infty) = F(\infty) + H(\infty). \quad (19)$$

Combining Eqs. (18) and (19) leads to:

$$\frac{m}{r} = \frac{CFR(\infty)}{1 - CFR(\infty)}, \quad (20)$$

where $\mathrm{CFR}(\infty) = F(\infty)/D(\infty)$ is the case fatality rate at the end of the epidemic wave. As the COVID-19 pandemic is still ongoing, we focus on the end of the first peak, where the number of active cases can be considered as ~0.

Socio-demographic data collection

Multiple sources were used for socio-demographic data. From the Social Science Research Council website [13] we obtained data on gender, race, population not between 18 and 65 (non-workforce), health insurance, infant and child mortality, life expectancy at birth, and GDP. Center for Disease Control and Prevention (CDC) data [14] was used for medical parameters - cardiovascular disease, cholesterol, hypertension, inactivity, smoking, consuming alcohol, obesity, cancer, chronic kidney disease, and chronic obstructive pulmonary disease (COPD). Census Reporter website [15] was used to obtain the percentage of the foreign population. Global Data Lab website [16] was used to obtain the Human Development Index (HDI) on the

subnational level. U.S. Census Bureau website [17] was used to obtain the median age, population density, and urban population proportion.

Pollution data collection

US environmental protection agency (EPA) Air Data service [18] data was used to obtain air quality measures, which were aggregated (on a daily level) for all listed cities. Pollutant species monitored consisted of gases (NO2, CO, SO2, O3), particulates (PM2.5 and PM10), Volatile Organic Compounds (VOC), NOx, and Hazardous Air Pollutants (HAP). [19] was used to obtain populations of cities used for weighting the averages during aggregation. In further analysis, yearly averages for each pollutant species (representing chronic pollution exposure) were used.

Weather data collection

The weather parameters were obtained from the NASA POWER project service [20] in an automated fashion using the POWER API and the custom Python scripts. Points of interest were coordinates obtained at Wikidata [21,22] for all the cities sorted by descending population size that comprises above 10% of the total country population. Parameters are listed in Table 1 and include temperature at 2m and 10m, measures of humidity and precipitation (wet bulb temperature, relative humidity, total precipitation), and insolation indices. The maximum daily predicted UV index was downloaded from OpenUV [23]. Weather parameters were then averaged for each USA state for the duration of the first peak.

Data transformation and principal component analysis

The distribution of most of the examined variables deviated from normality. To reduce skewness and the number of outliers in the data, appropriate transformations were applied (Table 1). Outliers are identified as values more than three scaled median absolute deviations (MAD) away from the median. After transformation, the remaining outliers were substituted by the transformed variable median value.

To reduce the number of variables, which was initially larger (62) than the sample size (51), we divided data into mutually related subsets and performed Principal Component Analysis (PCA) [24] on each group (Table 2). We grouped the variables according to the following two criteria: *i)* variables that present similar quantities, to allow easier interpretation of the principal components, *ii)* variables that are highly mutually correlated. Consequently, after PCA, correlations between the resulting predictors are reduced. Variables that did not satisfy the above criteria were not grouped, and they were used in the analysis as they are. Additionally, the variables that contributed to the relevant PCs in a way that was hard to interpret, were also treated as independent predictors. For example, the percentage of the youth population (Youth) has an opposite meaning from the other two age-related variables (Median age and percent of the population over 65), so that it was treated as an independent variable.

The number of PCs retained for each group was determined to explain >85% of the data variance. Afterward, a total of 29 variables (18 principal components and 11 independent variables) remained.

Data	Name (units)	Transformation f(x)
m/h	Morbidity	$x^{1/2}$
T2M	The mean temperature at 2m (°C)	$(x - (x)\ )^{1/3}$
$T2M_{MAX}$	The average maximal temperature at 2 meters (°C)	None
$T2M_{MIN}$	The average minimal temperature at 2 meters (°C)	$(x - (x)\ )^{1/3}$
T10M	The mean temperature at 10 meters	$x^{1/3}$
$T10M_{MAX}$	The average maximal temperature at 10 meters (°C)	None
$T10M_{MIN}$	The average minimal temperature at 10 meters (°C)	$(x - (x)\ )^{1/3}$

TS	The surface temperature (°C)	$log(x - min(x))$
T2MWET	The wet-bulb temperature at 2 meters (°C)	None
RH2M	Relative humidity at 2 meters (%)	$-\ log(max(x) - x)$
QV2M	Specific humidity at 2 meters (g/kg)	$log(x)$
T2MDEW	Dew point (°C)	None
PRECTOT	Precipitation (mm/day)	$x^{1/3}$
TQV	Total Column Precipitable Water (cm)	$log(x)$
UV	UV radiation index	$x^{1/2}$
ALLSKY_SFC_SW_DWN	All Sky Insolation Incident on a Horizontal Surface (MJ/m^2/day)	None
CLRSKY_SFC_SW_DWN	Clear Sky Insolation Incident on a Horizontal Surface (MJ/m^2/day)	$-\ log(max(x) - x)$
ALLSKY_SFC_LW_DWN	Downward Thermal Infrared (Longwave) Radiative Flux (MJ/m^2/day)	$log(x)$
PS	Pressure (mbar)	$-\ log(max(x) - x)$
WS2M	Wind speed at 2 meters (kts)	$-\ log(max(x) - x)$
WS10M	Wind speed at 10 meters (kts)	x^2
Elderly	Population over 65 (%)	None
Median age	Median age (years)	$log(x)$
Youth	Population under 18 (%)	$-\left((x)\ - x\right)^{1/2}$
Population density	Population density (people/km^2)	$log(x)$
BUAPC	Built-Up Area Per Capita (km^2/people)	$x^{-1/3}$
Urban Population	Urban population (%)	x^2
GDPpc	Gross Domestic Product per capita	$log(x)$
HDI	Human Development Index	$-\left((x)\ - x\right)^{1/2}$
Infant mortality	Infant mortality rate (per 1,000 live births)	$-\ log(x)$
Child mortality	Child mortality rate (per 1,000 live births)	$-\ log(x)$
Alcohol consumption	Adults' alcohol consumption binge drinking (%)	$log(x)$
Foreign-born population	Foreign-born population (%)	$log(x)$
Life expectancy	Life expectancy at birth (years)	$-\left((x)\ - x\right)^{1/2}$
Obesity	Obesity at age 20 and older (%)	None
CVD deaths	Age 65+ cardiovascular disease deaths (per 100,000 people)	$log(x)$
Hypertension	Adults with hypertension (%)	$log(x)$
High cholesterol	Population with high cholesterol (%)	$x^{1/2}$
Smoking	Population smoking (%)	None
Cardiovascular disease	Population with cardiovascular disease (%)	None
Diabetes	Population with diabetes (%)	$x^{1/2}$
Cancer	Population with cancer (%)	None
CKD	Population with chronic kidney disease (%)	$x^{1/3}$
COPD	Population with chronic obstructive pulmonary disease (%)	$log(x)$
Multiple chronic conditions	Population with multiple chronic conditions (%)	$log(x)$
Physical Inactivity	Physically inactive population (%)	$x^{1/2}$
White	Fraction of white in the population (%)	x^2
Black	Fraction of Afro-Americans in the population (%)	$x^{1/3}$
Latino	Fraction of Latino in the population (%)	$log(x)$
No Insurance Children	No health insurance under 18 (%)	$x^{1/2}$
No Insurance Adults	No health insurance 18-64 (%)	$x^{1/2}$
No Insurance Total	No health insurance all population (%)	$x^{1/2}$
No Insurance Black	No health insurance among black (%)	None
No Insurance Latino	No health insurance Latino (%)	None
No Insurance White	No health insurance white (%)	None

$PM_{2.5}$	$PM_{2.5}$ concentration (μg/m^3)	$-\left((x)\ -x\right)^{1/2}$
PM_{10}	PM_{10} concentration (μg/m^3)	$x^{1/3}$
O_3	O_3 concentration (ppm)	x^2
CO	CO concentration (ppm, 10^{-6})	None
SO_2	SO_2 concentration (ppb)	$x^{1/3}$
HAPs	Hazardous air pollutants concentration (μg/m^3)	$x^{1/3}$
NO_2	NO_2 concentration (ppb, 10^{-9})	$x^{1/3}$
NONOxNOy	Nitrous oxide concentration (ppb)	$x^{1/2}$

Table 1: List of variables with appropriate transformations.

Principal components	Variables
Temperature PC1	T2M, $T2M_{MAX}$, $T2M_{MIN}$, T10M, $T10M_{MAX}$, $T10M_{MIN}$, TS,T2MWET
Humidity PC1	RH2M, QV2M, T2MDEW
Precipitation PC1	PRECTOT, TQV
Radiation PC1-PC2	ALLSKY_SFC_SW_DWN, CLRSKY_SFC_SW_DWN, ALLSKY_SFC_LW_DWN
Wind Speed PC1	WS2M, WS10M
Seasonality PC1-PC2	Temperature PC1, Humidity PC1, Precipitation PC1, UV, Radiation PC2
Age PC1	Elderly, Median age
Density PC1-PC2	Population density, BUAPC, Urban population
Prosperity PC1-PC4	GDPpc, HDI, Infant Mortality, Child mortality, Alcohol consumption, Foreign-born, Life expectancy
Disease PC1-PC4	Obesity, CVD deaths, Hypertension, High Cholesterol, Smoking, Cardiovascular disease, Diabetes, Cancer, CKD, COPD, Multiple chronic conditions, Physical inactivity
No Insurance PC1-PC2	No Insurance Children, No Insurance Adults, No Insurance Total, No Insurance Black, No Insurance Latino, No Insurance White
NO PC1	NO_2, NONOxNOy

Table 2: Grouping of the variables before PCA and selected principal components from each group.

Relaxed LASSO regression

A modification of Lasso (Least Absolute Shrinkage and Selection Operator) [25] regression, Relaxed Lasso [26], was used to implement L1 regularization on high-dimensional data. Selected 29 variables were standardized before the first Lasso regression analysis. Hyperparameter λ was optimized by 5-fold cross-validation, with 40 dataset repartitions. 100 λ values in the range from 0 to the minimal λ value (which produces all zero terms) were put on the grid, where the optimal λ value was determined as having minimal MSE (Mean Squared Error) on the testing set. This hyperparameter value was used to train the first round model on the entire dataset. Only predictors with non-zero coefficients from this model were used in the second (relaxed) Lasso regression. The optimal λ value in the second round was determined by cross-validation as described for the first round, which was then used to train the final (second round) model on the entire dataset. By using Relaxed Lasso regression, noise from the high-dimensional data (in particular those variables that do not influence the output) is reduced, allowing for more accurate estimates of the reported regression coefficients. The final model from the second round was used for subsequent predictions, with its regression coefficients reported. This, and the other three procedures described below, were trained both with and without No Insurance (% of the uninsured population) data.

Relaxed Elastic net regression

Elastic net regression [27] was used for the implementation of L1 and L2 regularization. The procedure was similar to the Relaxed Lasso analysis explained above, only this time two hyperparameters – α and λ were optimized. These hyperparameters were put on a grid consisting of 100 uniformly distributed (from 0 to 1) α values, and 100 λ values chosen for each α value as described for the Lasso regression. Similarly,

as for Lasso, 5-fold cross-validation with 40 dataset repartitions was used. Optimal α and λ values were determined as those with minimal testing set MSE, which were used to train the first round model on the entire dataset. Predictors with non-zero coefficients from the final first-round model were used as an input for the second (relaxed) Elastic net round. Optimal hyperparameter (α and λ) values were determined by cross-validation equivalently as in the first round, which were then used to train the final (second round) model on the entire dataset. Regression coefficients obtained from the final model were reported, which was subsequently also used for predictions.

Random Forest and Gradient Boost

Ensembles of weak learners (decision trees) were implemented through Random Forest and Gradient Boost [28–31]. Optimal hyperparameters were determined by grid search, with 5-fold cross-validation and 40 dataset repartitions, equivalently to Lasso and Elastic net regressions. In each cross-validation round, input variables were preselected based on their significant correlations ($P < 0.1$ for either Pierson's, Spearman, or Kendall) with *m/r* on the testing set. This is to avoid overfitting by reducing the number of model predictors. For Random Forest, maximal number of splits, minimal leaf size and number of trained decision trees on the grid were respectively: {3, 6, 9, 12, 18, 20, 22, 24, 26, 30, 35, 37, 40, 43, 45, 47, 50}, {1, 2, …, 10}, {5, 8, 10, 14, 18, 26, 50, 106, 133, 160, 193, 226, 263, 300, 350, 400, 450, 500, 550, 600}. For Gradient Boost values of maximal number of splits, minimal leaf size, number of trained decision trees and learn rate on the grid were respectively: {1, 2, 3, 4, 5, 6, 7, 8, 10, 12, 14, 16, 24, 32}, {1, 2, 3, 4, 5, 8, 12, 16, 18, 20, 22, 25}, {5, 10, 13, 16, 21, 27, 38, 65, 92}, {0.1, 0.20, 0.25, 0.35, 0.5, 0.75, 1}. Combinations of the hyperparameter values that lead to the minimal testing set MSE were used to train the final models on the whole dataset. The input variable preselection in the final models was done on the entire dataset, equivalently as described above. Final models were used to estimate the predictor importance and in the predictions described below.

Predictions of δ(m/r)

Regression predictions of *δ(m/r)* were made by consensus, i.e., averaging the following final models described above: *i*) For chronic disease, population density, and pollution, all eight models were used (Lasso, Elastic net, Random Forest, Gradient Boost, each trained both with and without No Insurance). While R^2 for the decision tree based methods (Random Forest and Gradient Boost) is larger than for the linear regressions, the differences in the testing set MSE (prediction accuracy) were not large, so all eight models were used to achieve robust results. *ii*) For the percentage of population under 18 (Youth) the four models trained without No Insurance were used, as the strong correlation between Youth and No Insurance obscures the relation of Youth to *m/r. iii*) For the percentage of Afro-Americans (Black), the four nonparametric models (that can accommodate non-linear relations and interactions) were used (Gradient Boost and Random Forest both with and without No Insurance), as the contribution of this variable to *m/r* is not captured by linear regressions. All the averages above are weighted by 1/MSE so that models with higher prediction accuracy are included with larger weights.

References

[1] M.J. Keeling, P. Rohani, Modeling Infectious Diseases in Humans and Animals, Princeton University Press, Princeton, NJ, 2011.

[2] M. Djordjevic, A. Rodic, I. Salom, D. Zigic, O. Milicevic, B. Ilic, M. Djordjevic, A systems biology approach to COVID-19 progression in population, Adv Protein Chem Struct Biol. (2021). https://doi.org/10.1016/bs.apcsb.2021.03.003.

[3] M. Djordjevic, M. Djordjevic, B. Ilic, S. Stojku, I. Salom, Understanding Infection Progression under Strong Control Measures through Universal COVID-19 Growth Signatures, Global Challenges. (2021) 2000101. https://doi.org/10.1002/gch2.202000101.

[4] T. Britton, F. Ball, P. Trapman, A mathematical model reveals the influence of population heterogeneity on herd immunity to SARS-CoV-2, Science. 369 (2020) 846–849. https://doi.org/10.1126/science.abc6810.

[5] O. Diekmann, H. Heesterbeek, T. Britton, Mathematical Tools for Understanding Infectious Disease Dynamics, Princeton University Press, 2012. https://doi.org/10.1515/9781400845620.

[6] G.N. Wong, Z.J. Weiner, A.V. Tkachenko, A. Elbanna, S. Maslov, N. Goldenfeld, Modeling COVID-19 Dynamics in Illinois under Nonpharmaceutical Interventions, Phys. Rev. X. 10 (2020) 041033. https://doi.org/10.1103/PhysRevX.10.041033.

[7] I. Salom, A. Rodic, O. Milicevic, D. Zigic, M. Djordjevic, M. Djordjevic, Effects of Demographic and Weather Parameters on COVID-19 Basic Reproduction Number, Frontiers in Ecology and Evolution. 8 (2021) 524. https://doi.org/10.3389/fevo.2020.617841.

[8] M. Djordjevic, I. Salom, S. Markovic, A. Rodic, O. Milicevic, M. Djordjevic, Inferring the Main Drivers of SARS-CoV-2 Global Transmissibility by Feature Selection Methods, GeoHealth. 5 (2021) e2021GH000432. https://doi.org/10.1029/2021GH000432.

[9] O. Milicevic, I. Salom, A. Rodic, S. Markovic, M. Tumbas, D. Zigic, M. Djordjevic, M. Djordjevic, PM2.5 as a major predictor of COVID-19 basic reproduction number in the USA, Environ. Res. 201 (2021) 111526. https://doi.org/10.1016/j.envres.2021.111526.

[10] B.F. Maier, D. Brockmann, Effective containment explains subexponential growth in recent confirmed COVID-19 cases in China, Science. 368 (2020) 742–746. https://doi.org/10.1126/science.abb4557.

[11] F. Brauer, Compartmental Models in Epidemiology, in: F. Brauer, P. van den Driessche, J. Wu (Eds.), Mathematical Epidemiology, Springer Berlin Heidelberg, Berlin, Heidelberg, 2008: pp. 19–79. https://doi.org/10.1007/978-3-540-78911-6_2.

[12] M. Martcheva, An Introduction to Mathematical Epidemiology, Springer, Boston, MA, 2015. doi: 10.1007/978-1-4899-7612-3.

[13] Measure of America, Mapping America: Demographic Indicators. http://measureofamerica.org/tools-old/, 2018 (accessed 10 July 2021).

[14] CDC, CDC - Behavioral Risk Factor Surveillance System. https://www.cdc.gov/brfss/index.html, 2019 (accessed 10 July 2021).

[15] U.S. Census Bureau, Nativity in the United States American Community Survey 1-year estimates. https://censusreporter.org/, 2019 (accessed 10 July 2021).

[16] J. Smits, I. Permanyer, The Subnational Human Development Database, Scientific Data. 6 (2019) 190038. https://doi.org/10.1038/sdata.2019.38.

[17] U.S. Census Bureau, Population Division, Annual Estimates of the Resident Population by Single Year of Age and Sex for the United States, States, and Puerto Rico Commonwealth: April 1, 2010, to July 1, 2018. https://www.census.gov/topics/population.html, 2019 (accessed 10 July 2021).

[18] US Environmental Protection Agency, Air Quality System Data, US EPA. https://www.epa.gov/outdoor-air-quality-data, 2020 (accessed 10 July 2021).

[19] US Census Bureau, City and Town Population Totals: 2010-2019, The United States Census Bureau. https://www.census.gov/data/tables/time-series/demo/popest/2010s-total-cities-and-towns.html, 2020 (accessed 7 October 2021).

[20] NASA Langley Research Center, The Prediction of Worldwide Energy Resources (POWER) Project. https://power.larc.nasa.gov/, 2020 (accessed 10 July 10, 2021).

[21] Wikipedia, List of United States cities by population. https://en.wikipedia.org/w/index.php?title=List_of_United_States_cities_by_population&oldid=1017904123, 2021 (accessed 10 July 2021).
[22] Wikipedia, List of states and territories of the United States by population. https://en.wikipedia.org/w/index.php?title=List_of_states_and_territories_of_the_United_States_by_population&oldid=1016990633, 2021 (accessed 10 July 2021).
[23] OpenUV, Global UV Index API. https://www.openuv.io/, 2020 (accessed 10 July 2021).
[24] I.T. Jolliffe, Principal Component Analysis, 2nd ed., Springer-Verlag, New York, 2002. https://doi.org/10.1007/b98835.
[25] R. Tibshirani, Regression Shrinkage and Selection Via the Lasso, Journal of the Royal Statistical Society: Series B (Methodological). 58 (1996) 267–288. https://doi.org/10.1111/j.2517-6161.1996.tb02080.x.
[26] N. Meinshausen, Relaxed Lasso, Computational Statistics & Data Analysis. 52 (2007) 374–393. https://doi.org/10.1016/j.csda.2006.12.019.
[27] H. Zou, T. Hastie, Regularization and Variable Selection via the Elastic Net, Journal of the Royal Statistical Society. Series B (Statistical Methodology). 67 (2005) 301–320.
[28] L. Breiman, Random Forests, Machine Learning. 45 (2001) 5–32. https://doi.org/10.1023/A:1010933404324.
[29] L. Breiman, Bagging predictors, Machine Learning. 24 (1996) 123–140. https://doi.org/10.1007/BF00058655.
[30] Y. Freund, R.E. Schapire, A Decision-Theoretic Generalization of On-Line Learning and an Application to Boosting, Journal of Computer and System Sciences. 55 (1997) 119–139. https://doi.org/10.1006/jcss.1997.1504.
[31] T. Hastie, R. Tibshirani, J. Friedman, The Elements of Statistical Learning, 2nd ed., Springer, New York, 2009.
